# Biological Control of *Fusarium culmorum*, *Fusarium graminearum* and *Fusarium poae* by Antagonistic Yeasts

**DOI:** 10.3390/pathogens11010086

**Published:** 2022-01-11

**Authors:** Izabela Podgórska-Kryszczuk, Ewa Solarska, Monika Kordowska-Wiater

**Affiliations:** 1 Department of Analysis and Food Quality Assessment, University of Life Sciences in Lublin, Skromna 8, 20-704 Lublin, Poland; izabela.podgorska-kryszczuk@up.lublin.pl; 2 Department of Biotechnology, Microbiology and Human Nutrition, University of Life Sciences in Lublin, Skromna 8, 20-704 Lublin, Poland; ewa.solarska@up.lublin.pl

**Keywords:** *Fusarium culmorum*, *Fusarium graminearum*, *Fusarium poae*, biological control, yeast, volatile organic compounds, chitinase, β-1,3-glucanase, siderophores

## Abstract

The genus *Fusarium* is considered to be one of the most pathogenic, phytotoxic and toxin-producing group of microorganisms in the world. Plants infected by these fungi are characterized by a reduced consumer and commercial value, mainly due to the contamination of crops with mycotoxins. Therefore, effective methods of reducing fungi of the genus *Fusarium* must be implemented already in the field before harvesting, especially with alternative methods to pesticides such as biocontrol. In this study we identified yeasts that inhibit the growth of the pathogenic fungi *Fusarium culmorum*, *F. graminearum* and *F. poae*. Tested yeasts came from different culture collections, or were obtained from organic and conventional cereals. The greater number of yeast isolates from organic cereals showed antagonistic activity against fungi of the genus *Fusarium* compared to isolates from the conventional cultivation system. *Cryptococcus carnescens* (E22) isolated from organic wheat was the only isolate that limited the mycelial growth of all three tested fungi and was the best antagonist against *F. poae*. Selected yeasts showed various mechanisms of action against fungi, including competition for nutrients and space, production of volatile metabolites, reduction of spore germination, production of siderophores or production of extracellular lytic enzymes: chitinase and β-1,3-glucanase. Of all the investigated mechanisms of yeast antagonism against *Fusarium*, competition for nutrients and the ability to inhibit spore germination prevailed.

## 1. Introduction

Filamentous fungi and their toxic metabolites significantly reduce the quality of food and feed and pose a serious threat to food safety. Mycotoxins cause biochemical, physiological and pathological changes in living organisms and produce a toxic effect even at low concentrations [1]. Fungi of the genus *Fusarium* are the most frequently isolated pathogens from important agricultural plants, including wheat [2] and maize [3]. They commonly occur in soil, plant debris and various organic substrates, where they live as saprophytes, feeding on dead organic matter. Due to their high pathogenicity and toxigenicity, they cause huge economic losses [4]. Fungi of the genus *Fusarium* have the ability to synthesize numerous toxic metabolites, including zearalenone, trichothecenes, fumonisins, moniliformin, and beauvericin. These toxins accumulate in plant cells, with which they get into the human and animal food chains, becoming the cause of serious human and animal diseases [4,5,6]. *Fusarium* mycotoxins are mainly produced by *F. culmorum*, *F. graminearum*, *F. poae*, *F. avenaceum*, *F. oxysporum*, *F. sporotrichioides*, and *F. verticillioides* [5,7].

Pathogenic fungi and their toxic metabolites pose a risk to food health safety. There is a high demand for new solutions, aiming to reduce the entry of mycotoxins into the food chain. Research results indicate that most *Fusarium* mycotoxins are stable and remain intact at the stage of food processing [8]. This means that limiting the presence of toxin-producing fungi in grain is extremely important, because the concentration of toxins in flour contaminated with mycotoxins and in products, such as bread, will be similar. During baking process no significant degradation of these compounds will take place [9,10]. In response to this challenge, there is a growing interest in biological methods of controlling fungal contamination in the field, including the use of microorganisms, and there is ongoing search for new antifungal starter cultures in various branches of the food industry [11,12]. In the literature, the use of biological control agents has been documented as a potential alternative to control *Fusarium* spp. [13]. 

One group of fungal biocontrol agents that has recently been attracting increased attention from scientists and industry is yeast. Yeast occurs on the above-ground and underground parts of plants and uses various mechanisms to effectively prevent the development of *Fusarium* spp. both during the growing season and in the storage [14]. The main mechanisms of yeast action that play a key role in the biological control of pathogenic fungi are (1) competition for nutrients and space; (2) production of antifungal diffusible and volatile metabolites; (3) secretion of enzymes that degrade the fungal cell wall; and (4) the secretion and release of antimicrobial compounds, such as killer toxins or “mycocins” [15,16]. A special case of competition between yeast and fungi is competition for iron, which is necessary for the cellular iron metabolism. This mechanism is based on the synthesis of siderophores-compounds which chelate iron ions from an environment which has a low iron availability [17,18]. The above-mentioned mechanisms of action against fungi are usually used simultaneously, thereby enhancing the antagonistic effect. Unfortunately, it is often difficult to select a single strain with a broad spectrum of activity against many pathogens. Therefore, compatible strains are sought to ensure the necessary differentiation of action. The use of a mixture of antagonists has certain advantages such as increasing their effectiveness and broadening their efficacy [19]. 

The aim of this study was to select and identify yeast isolates which showed the potential to inhibit *Fusarium* species, to investigate their mechanisms of action, and to use them for the protection of wheat grain.

## 2. Results

### 2.1. Isolation of Yeast from Cereals

In this study, samples were taken from organic and conventional farming fields, but the number of isolated yeasts differed significantly between the agricultural systems. After isolation of pure cultures, 17 morphologically diverse isolates of yeast and yeast-like fungi were isolated from plants grown using conventional methods, and 34 from organic plants. The largest amount of yeast was isolated from ears of corn, which accounted for 61.8% (*n* = 21) and 58.8% (*n* = 10) of all the yeast isolated from organic and conventional cereals, respectively. 

### 2.2. Confrontation Assay

Twenty eight yeast isolates inhibited the mycelial growth of *F. culmorum*, *F. graminearum*, and *F. poae* in a direct confrontation assay. Twelve of these yeasts came from a culture collection, and 16 from the natural environment—5 from conventionally grown cereals and 11 from organic cereals (Table 1). 

Seven most effective strains were selected and used in further studies. They included 3 collection strains: *Candida shehatae* ATCC 22984 (C13), *Candida fluviatilis* CBS 6776 (C14), *Candida tropicalis* DSM 7524 (C28), and four isolates from the natural environment: K2, K10, E20, E22. These seven isolates inhibited the mycelial growth of at least one of the pathogens, creating an inhibition zone larger than 5 mm. The yeast isolates C13, C14, C28, K10 and E20 limited the growth of *F. culmorum* and *F. graminearum*, but did not inhibit the growth of *F. poae*. The yeast designated as K2 restricted the growth of *F. graminearum* and *F. poae*, but did not affect *F. culmorum*. The only yeast that limited the growth of all 3 test pathogenic fungi was E22, an isolate from organic cereals.

### 2.3. Yeast Identification

Based on morphological characteristics and comparison of nucleotide variations in the internal transcribed spacer (*ITS*) region of the tested isolates with the sequences available in the NCBI GeneBank database, the four most effective wild yeast isolates were identified as:

K2—*Meyerozyma guilliermondii*

K10—*Cyberlindnera saturnus*

E20—*Rhodotorula glutinis*

E22—*Cryptococcus carnescens*

### 2.4. Effect on Spore Germination of Fusarium In Vitro

**Figure 1:**
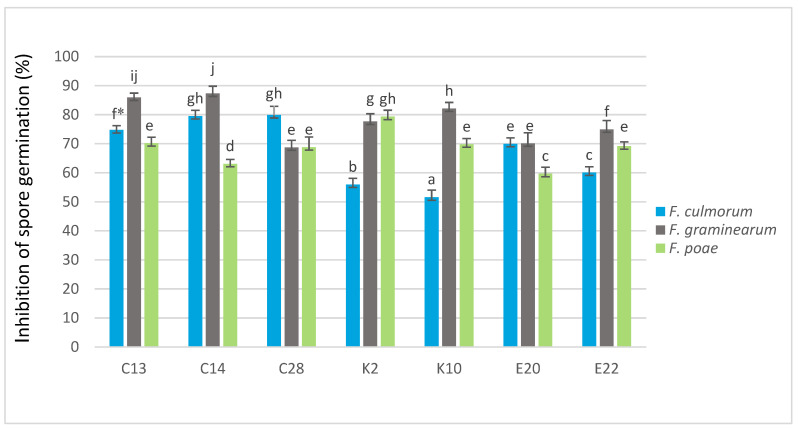
Inhibition of *F. culmorum*, *F. graminearum* and *F. poae* spore germination (%) by antagonistic yeasts. The fungal and yeast isolates were co-cultured in wort broth medium at 25 °C for 20 h. Error bar represents the standard error. * Values marked with the same letters do not differ significantly at *p* < 0.05 (Tukey’s post hoc test).

All of the seven test yeasts inhibited the germination of *F. culmorum*, *F. graminearum* and *F. poae* spores compared to the control (Figure 1). The isolates C28 and C14 were the most effective against *F. culmorum*, reducing spore germination by 79.86% and 79.52%, respectively. On the other hand, the yeast isolates K10 and K2 were the weakest inhibitors of the spore germination (51.54% and 55.97%, respectively). The isolates C14 and C13 were found to be the most effective in reducing the germination of *F. graminearum* spores (87.29% and 85.91%, respectively). The weakest inhibitory effect on the spore germination of *F. graminearum* was observed for the yeasts C28 (68.73%) and E20 (70.1%). K2 was the most effective inhibitor of *F. poae* spore germination (79.32%). The weakest inhibitory effect on the spore germination of this pathogen was recorded during its co-cultivation with the yeast isolates E20 (59.66%) and C14 (63.05%).

### 2.5. Production of Volatile Organic Compounds

**Figure 2:**
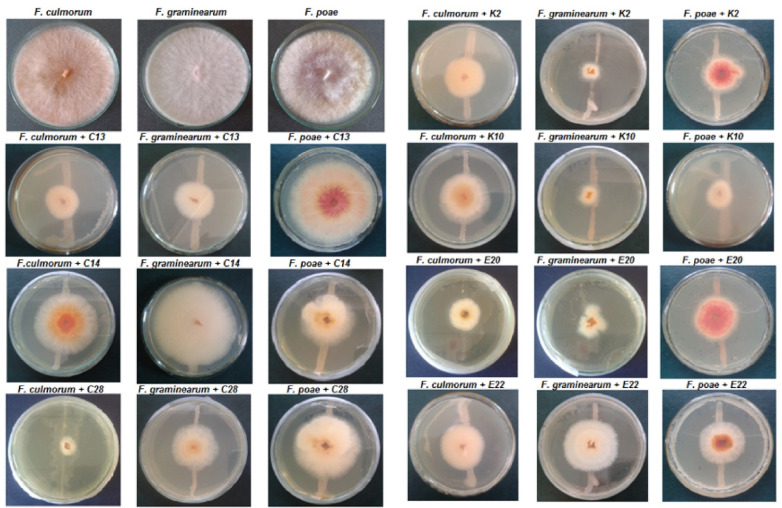
Dual-culture assay for bioactivity of volatile organic compounds produced by the test yeasts after 7 days of incubation at 28 °C on malt extract agar.

All of the tested yeasts inhibited the growth of the mycelia of *F. culmorum*, *F. graminearum* and *F. poae* compared to the control. The results are presented in Table 2 and in Figure 2.

The yeasts C28 and C13 were the strongest inhibitors of *F. culmorum* growth, causing an 87.59% and an 83.70% reduction in the mycelial surface, respectively. The most effective reduction in the growth of *F. graminearum* was observed during incubation with the isolates K10 (87.67%) and K2 (86.11%). The growth of *F. poae* mycelium was limited to the greatest extent by the yeast K10 (an 80.93% reduction), followed by E22 (a 70% reduction). It was found that all the environmental yeasts were more effective in inhibiting the growth of *F. graminearum* and *F. poae* compared to the yeasts from the culture collection. 

### 2.6. Enzymatic Activities 

**Figure 3:**
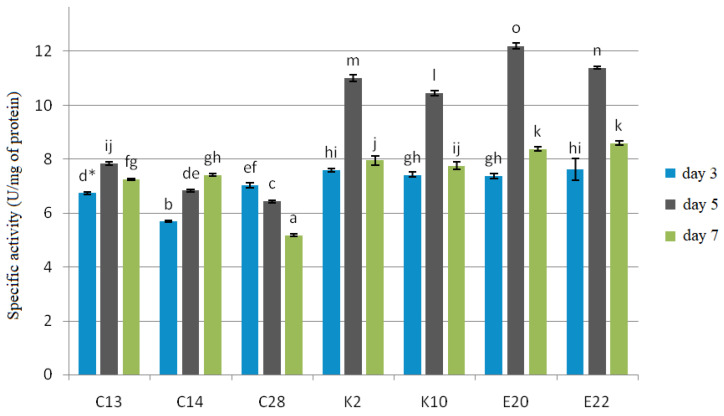
Specific activity of chitinase on the 3rd, 5th and 7th day of cultivation with *F. culmorum*, *F. graminearum* and *F. poae* cell wall preparations in the medium as a carbon source. Error bar represents the standard error. * Values marked with the same letters do not differ significantly at *p* < 0.05 (Tukey’s post hoc test).

**Figure 4:**
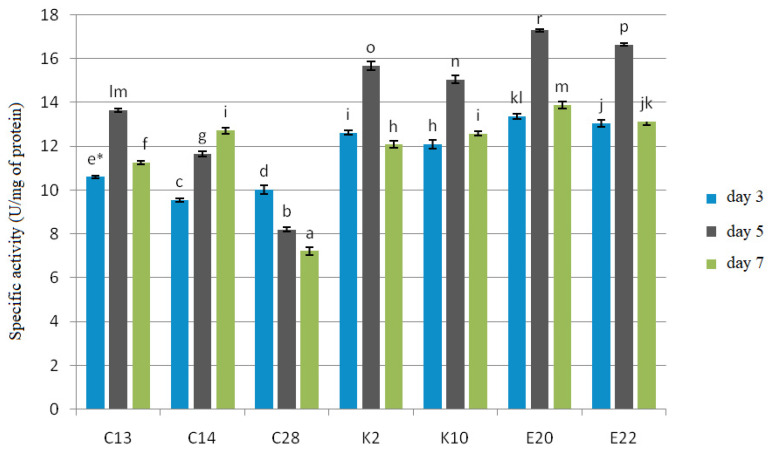
Specific activity of β-1,3-glucanase on the 3rd, 5th and 7th day of yeast cultivation with *F. culmorum*, *F. graminearum* and *F. poae* cell wall preparations in the medium as a carbon source. Error bar represents the standard error. * Values marked with the same letters do not differ significantly at *p* < 0.05 (Tukey’s post hoc test).

All the test yeasts produced chitinase and β-1,3-glucanase after 3, 5 and 7 days of cultivation with cell wall preparations (CWP) of *F. culmorum*, *F. graminearum* and *F. poae* in the medium as a carbon source (Figure 3 and Figure 4).

The experiment showed that β-1,3-glucanase had a more specific activity, ranging from 7.22 to 17.30 U/mg protein, than chitinase, whose specific activity ranged from 5.17 to 12.21 U/mg protein. The best producers of both enzymes were the yeasts E20 and E22, for which the test enzymes reached their maximum specific activities on the 5th day of cultivation: 12.21 U/mg protein and 11.93 U/mg protein for chitinase, and 17.30 U/mg protein and 16.95 U/mg protein for β-1,3-glucanase, respectively. The yeast isolates C13, K2, K10 also showed the maximum enzyme activity on the 5th day of cultivation, which for chitinase was 7.84 U/mg protein, 11.00 U/mg protein, and 10.44 U/mg protein, respectively, and for β-1,3-glucanase—13.65 U/mg protein, 15.68 U/mg protein, and 15.06 U/mg protein, respectively. C28 reached the maximum enzyme activity on the 3rd day of cultivation, which was 7.03 U/mg protein for chitinase and 10.02 U/mg protein for β-1,3-glucanase, and in the following days the enzymatic activity was gradually falling. The yeast isolate C14 showed the maximum activity of 7.41 U/mg protein for chitinase and 12.75 U/mg protein for β-1,3-glucanase on the 7th day of cultivation.

### 2.7. Siderophore Production

**Figure 5:**
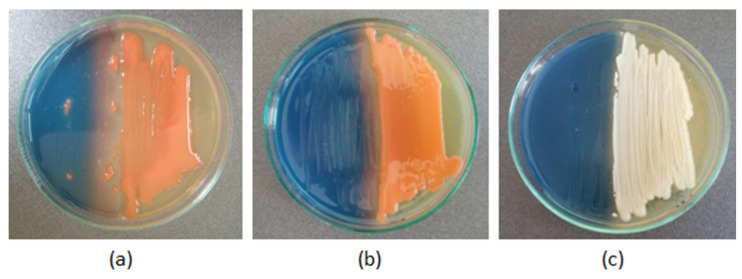
Results of the universal test for the detection of siderophores using CAS agar after 14 days of incubation at 28 °C: (**a**) control—discoloration of CAS agar to orange, evidencing the production of siderophores by the yeast *Rhodotorula glutinis*; (**b**) discoloration of CAS agar by the test yeast E20; (**c**) no discoloration of agar by the test yeast C13.

In the present study, the ability to produce siderophores was identified in only one of the yeast isolates—E20, isolated from organic cereals (Figure 5b). The remaining yeasts did not cause discoloration of the CAS medium after 14 days of incubation.

### 2.8. Antagonistic Activities on Wheat Grain

**Figure 6:**
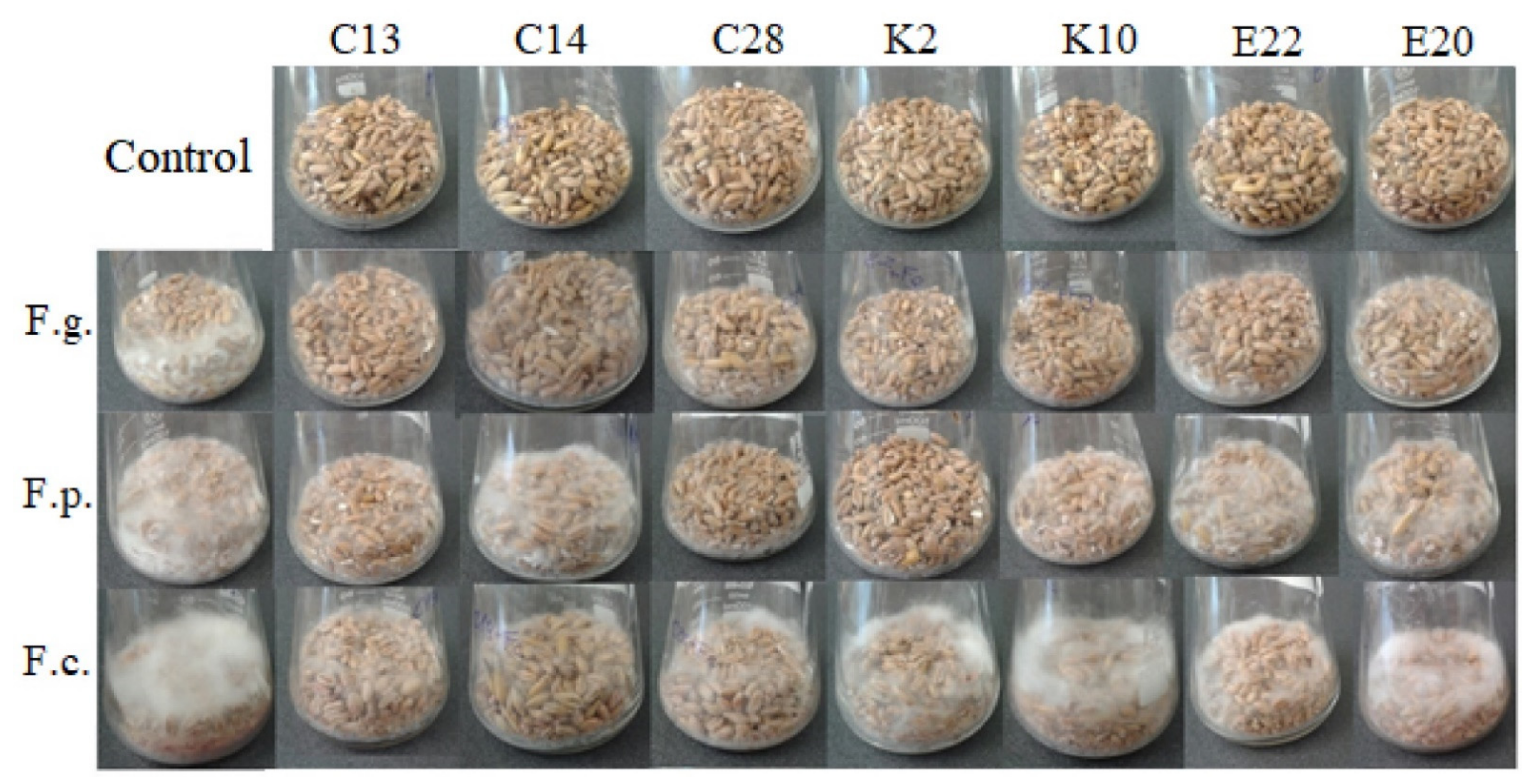
Wheat grain after a 14-day incubation (28 °C) of *F. culmorum*, *F. graminearum* and *F. poae* with the test yeasts and the controls. F.g.—*F. graminearum*, F.p.—*F. poae*, F.c.—*F. culmorum*.

A visual assessment of the effect of the test yeasts on the growth of *F. culmorum*, *F. graminearum* and *F. poae* on wheat grain was performed after 7 and 14 days of incubation at 28 °C. The control grain was moderately overgrown with *F. culmorum*, *F. graminearum* and *F. poae* mycelium after 7 days of incubation at 28 °C, but after 14 days strong growth was observed. *F. graminearum* showed the slowest growth after incubation with the test yeasts on wheat grain, and no growth was observed for this fungal species in any of the variants of the experiment after 7 days. After 14 days, a slight growth of *F. graminearum* in the presence of the yeasts C14, C28, K2, E20 and E22 was noted. *F. culmorum* showed poor growth after 7 and 14 days of incubation with the isolates C13 and C14, but the mycelium of this pathogen grew strongly on wheat grain in the presence of the yeasts C28, K2, K10, E20 and E22. The growth of *F. poae* after 7 and 14 days of incubation with the test yeasts was inhibited only by the isolates C28 and K2 (Figure 6).

## 3. Discussion

Contamination of food with fungi is a serious problem, which can occur at various stages of the food production chain, at harvesting, in storage and during processing. The growth of fungi leads to quality changes in food, but it can also have negative health effects as some fungi produce mycotoxins. Infection by fungi of the genus *Fusarium*, and thus the presence of mycotoxins in plant raw material, is a frequent problem, but the prevention of these pathogens is difficult, even if good agricultural practices are maintained. The use of microorganisms as biological control agents for the protection of crops is not a new approach, but the growing interest in this method leads to the intensification of research efforts worldwide. In the presented study it was assumed that the yeast should come from an environment in which it could potentially be used to protect cereal grains and products made from them against pathogenic fungi. In most biocontrol researches, potential antagonists are usually isolated from symptomless plants [20]. The same number of wheat, barley and oats samples were collected from the fields of organic and conventional cultivation, but the number of yeast isolates differed significantly between the systems. Other studies reported an increase in the number and diversity of microorganisms in plants and soil from the organic farming system compared to conventional [21,22]. The reduction in numbers and diversity of microorganisms in a conventional cultivation system can be explained by the long-term stress caused by the use of synthetic fertilizers and chemical plant protection products that can eliminate certain groups of microorganisms, including yeasts [23].

The yeast isolates selected in this study showed similar activity against the tested pathogens, but only *Cryptococcus carnescens* (E22) isolated from the organic wheat limited the mycelial growth of all three fungi *F. culmorum*, *F. graminearum*, and *F. poae*. In recent years yeast from the genus *Cryptococcus* has been one of the most commonly investigated microorganisms for the control of *Fusarium* spp. [14]. Rong and McSpadden Gardener [24] reported that yeast *Cryptococcus flavescens* strain OH182.9_3C shows a high efficiency to control *Fusarium* head blight of wheat. Biological control of *Fusarium* head blight was demonstrated also for yeast strains *Cryptococcus* sp. OH 71.4, *C. nodaensis* OH 182.9, and *Cryptococcus* sp. OH 181.1 [25]. Yeasts antagonists can employ various biocontrol mechanisms such as competition, antibiosis, and the production of cell-wall degrading enzymes. Unfortunately, it is difficult to select a single broad-spectrum strain against multiple pathogens of the genus *Fusarium*, therefore in this study compatible strains are sought [26].

A key factor in the biological activity of yeast against pathogenic fungi is the competition for nutrients and space [17,27]. Therefore, in this study, yeast isolates were co-cultured with pathogenic fungi as the first stage in the selection of the most effective inhibitors of the growth of *F. culmorum*, *F. graminearum* and *F. poae*. After 7 days of incubation, only twenty-eight of one hundred yeasts inhibited the mycelial growth of at least one of the pathogens. For further research, we selected isolates which formed the largest growth inhibition zones: *C. shehatae* (C13), *C. fluviatilis* (C14), *C. tropicalis* (C28), *M. guilliermondii* (K2), *C. saturnus* (K10), *R. glutinis* (E20) and *C. carnescens* (E22). It has been demonstrated that yeasts, as biological control agents, effectively compete with fungi for space by occupying wound sites on the plant and using nutrients, thereby displacing the pathogen [16,28]. Yeast is able to quickly assimilate glucose, fructose or sucrose, while preventing the growth of undesirable microorganisms [15]. Vero et al. [27] found that competition for nutrients and space was the main mode of action of the antagonistic yeasts *Cryptococcus laurentii* and *Candida ciferrii* against *Penicillium* spp. Both yeast species inhibited the growth of the pathogen on apples, however, no metabolites with fungicidal properties were detected in any of the yeast cultures and no enzymatic activity was observed. The analysis of sugars contained in apple wounds showed a decrease in the concentration of sucrose and glucose during incubation, and the addition of amino acids increased the yeast population.

Another antagonistic mechanism that yeast may use against fungi is the inhibition of fungal spore germination. This ability can be very important for controlling fungi in the soil and in post-harvest conditions [29]. The literature shows that the ability of yeast to inhibit the germination of spores is determined by various mechanisms. Yeast quickly colonizes the surface of the plant, displacing pathogenic fungi and preventing their spore germination. Various yeasts can also produce different types of antibiotic agents, e.g., rhodotorulic acid, heptadecanoic acid, methylheptadecanoic acid, or aureobazidin A, as well as volatile metabolites and enzymes that prevent the germination of pathogen spores [30,31,32]. The ability of yeasts to inhibit fungal spore germination was confirmed by Zhang et al. [33], who used *Pichia fusiformata*, *Metschnikowia* sp. and *Aureobasidium pullulans* to reduce *Monilinia laxa*. In the present study, all the selected yeasts were able to inhibit the spore germination of *F. culmorum*, *F. graminearum*, and *F. poae* fungi by 51.54% to 87.29%. In general, the greatest reduction in fungal spore germination was obtained in cultures of pathogens with C13, C14 and C28 yeasts, and *F. culmorum* was the most sensitive. Yeasts cells located in close proximity to hyphae or mycelium aggregates suggest that the main mechanism of action may be competition for space and nutrients. 

One of the mechanisms by which yeast reduce the growth of *Fusarium* fungi is the production of volatile organic compounds. In this study it was found that all the test isolates inhibited the growth of *F. culmorum*, *F. graminearum* and *F. poae* mycelium after 7 days of co-incubation in tightly closed Petri dishes. The degree of inhibition of pathogen growth differed among isolates of yeast, with several of the isolates inhibiting fungal growth by more than 80%. Overall, the K10, E20 and K2 strains were the best producers of volatile compounds. The yeast that stood out in this mechanism of inhibition was the K10 strain, which inhibited in vitro two species of *F. graminearum* and *F. poae* by 87.67 ± 0.59% and 80.93 ± 0.85%, respectively. To identify volatile metabolites produced by yeast more research needs to be conducted, but literature reports indicate that yeasts produce several classes of these metabolites with a fungal inhibitory effect: alcohols, esters, alkanes, alkenes, arenes, organic acids, ketones, aldehydes, and amines [34,35,36]. The antimicrobial properties of alcohols have been known for a long time and are used in the preparation of disinfectants and preservatives. Their main site of action seems to be in the plasma membrane, where the accumulation of solvents can affect the organization and stability of the lipid bilayer. Alcohols increase the permeability of the membrane, accelerating the passive diffusion of ions and metabolites [37].

Another mechanism that yeasts use to reduce the growth of pathogenic fungi is the production of enzymes that degrade the fungal cell wall [38]. The capacity of diverse antagonistic microorganisms to produce chitinases [39] and β-1,3-glucanase [40] in particular is widely known. Microbial production of enzymes depends on many factors: primarily, the strain used, but also the carbon source in the culture medium, culture conditions and duration [41]. The type of medium used is an important factor as the medium provides nutrients for the microorganisms, and the use of different culture media often produces different results. In this study, CWP of *F. culmorum*, *F. graminearum* and *F. poae* were used in the yeast culture medium as the only carbon source. The literature confirms that CWP are good inducers of enzyme production [40]. In this present study, all the test yeast isolates produced both chitinase and β-1,3-glucanase, and the isolates *R. glutinis* (E20) and *C. carnescens* (E22) isolated from organic cereals were the best producers of these enzymes. Masih and Paul [42], who examined the ability of *Pichia membranifaciens* to produce β-1,3-glucanase, found that the yeast secreted the highest amounts of this enzyme in the presence of a *Botrytis cinerea* CWP. Chan and Tian [43], in their study on the ability of *Cryptococcus albidus* and *P. membranifaciens* to produce exo- and endo-chitinases, also proved that CWP of fungi *Monilinia fructicola* and *Penicillium expansum* were good inducers of the secretion of these enzymes. Similarly, Lopes et al. [44] found a positive effect of the addition of a *Colletotrichum acutatum* CWP to the medium on the production of chitinase and β-1,3-glucanase by the yeast *Saccharomyces cerevisiae*.

## 4. Materials and Methods

### 4.1. Microorganisms

The phytopathogenic fungi *F. culmorum*, *F. graminearum*, and *F. poae* were obtained from the Culture Collection of the Department of Biotechnology, Microbiology and Human Nutrition of the University of Life Sciences in Lublin, Poland. Forty nine strains of yeasts belonging to 9 genera: *Candida*, *Rhodotorula*, *Saccharomyces*, *Kluyveromyces*, *Hansenula*, *Pichia*, *Pachysolen*, *Yarrowia*, and *Trichosporon* were obtained from the same Culture Collection, previously purchased from different culture collections: Japan Collection of Microorganisms (JCM), American Type Culture Collection (ATCC), German Collection of Microorganisms and Cell Cultures (DSMZ), Centraalbureau voor Schimmelcultures (CBS), ARS Culture Collection (NRRL). Another 51 yeast strains were isolated from grains, ears, stems and roots of wheat, oat and barley grown using conventional and organic farming methods in Eastern Poland. The cultures of fungi and yeasts were kept fresh and viable by periodical transfers on malt extract agar medium (30 g malt extract, 5 g mycological peptone, 15 g agar per 1 L of distilled water) under aseptic conditions throughout the study. Strains were stored at 4 °C for routine cultivation.

### 4.2. Yeast Isolation and Confrontation Assay

Five grams of each part of the cereal plants were placed in flasks containing 45 mL sterile Ringer solution and kept on a rotary shaker (Infors HT Minitron, Infors AG, Bottmingen, Switzerland) at 180 rpm for 10 min. Serial dilutions of these suspensions were transferred into Petri dishes containing chloramphenicol yeast glucose agar (BTL, Lodz, Poland). After 48 h incubation at 28 °C, single colonies of yeast and yeast-like fungi were selected and spread onto malt extract agar medium.

**Figure 7:**
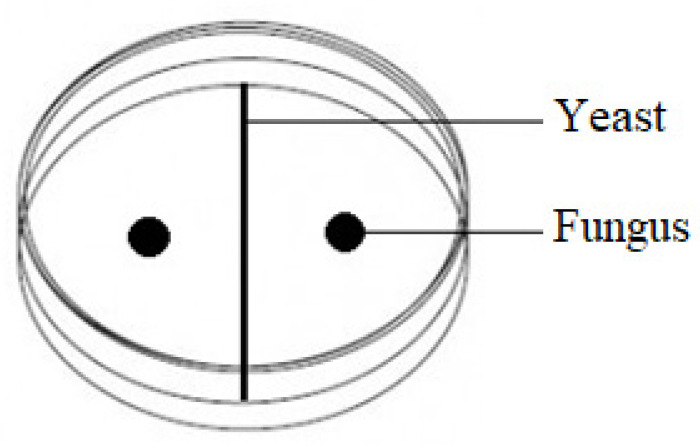
Scheme of the inoculation of microorganisms in the direct confrontation assay.

Inhibition of the mycelial growth of *F. culmorum*, *F. graminearum* and *F. poae* by the test yeasts was assessed in Petri dishes containing 20 mL malt extract agar. For the direct confrontation assay, a 2-day-old yeast culture was inoculated in the center of a plate, and pathogen mycelial plugs (5 mm in diameter) corked from a 7-day-old culture were placed symmetrically (25 mm away) on both sides of the yeast (Figure 7). The control was a Petri dish on which physiological sodium chloride solution was applied instead of the yeast. Three replicates were used for each combination of the experiment. After 7 days at 28 °C, the mycelial growth inhibition zone near the yeast was measured. The yeasts that most effectively inhibited the growth of *F. culmorum*, *F. graminearum* and *F. poae* mycelia were selected for further studies.

### 4.3. Yeast Identification 

The yeasts from the natural environment which had been the most effective inhibitors of the mycelial growth of *F. culmorum*, *F. graminearum* and *F. poae* in the direct confrontation assay were originally identified by morphological characteristics (macro- and microscopic observations). Morphological identification has been strengthened by the analysis of the *ITS* gene region. DNA was isolated from pure cultured cells using Genomic Mini AX Yeast DNA extraction kit (A&A Biotechnology, Gdańsk, Poland), according to the manufacturer’s protocol. Partial amplification of the ITS region was performed using the universal primers ITS1 (50-TCCGTAGGTGAACCTGCGG-30) and ITS4 (50-TCCTCCGCTTATTGATATGC-30) [45]. Amplification was performed in a gradient thermocycler (Bio-Rad, Warsaw, Poland) in a final volume of 50 μL containing 20 pmol of each primer (ITS-1 and ITS-4), 25 µL 2 × PCR Master Mix (A&A Biotechnology, Gdańsk, Poland), and 2 μL of DNA sample. The mixture was first denatured at 95 °C for 5 min. Then, 40 cycles of PCR were performed with denaturation at 95 °C for 1 min, annealing at 55 °C for 30 s, and extension at 72 °C for 30 s. At the end of the last cycle, the mixture was incubated at 72 °C for 10 min. The PCR products were separated by electrophoresis in a 1.5% (w/v) agarose gel with PCR 100 bp Low DNA ladder (Sigma-Aldrich, Poznań, Poland) as a molecular size standard, and stained with ethidium bromide. After electrophoresis, the PCR products were visualized under UV light using the GelDoc 2000 gel documentation system (BioRad, Hercules, CA, USA). The PCR products were sequenced by Genomed (Warsaw, Poland). The sequences obtained were analyzed with Finch TV 1.4.0 software (Geospiza Inc., Seattle, WA, USA). They were then combined using GeneDoc 2.7.000 [46] and compared to the National Center for Biotechnology Information (NCBI) database using the basic local alignment search tool (BLAST).

### 4.4. Effect on Pathogen Spore Germination In Vitro 

The effect of the antagonistic yeasts on *F. culmorum*, *F. graminearum* and *F. poae* spore germination was assessed in wort broth medium (15 g malt extract, 1 g peptone, 12.5 g maltose, 2.5 g glucose, 1 g K_2_HPO_4_, and 1 g NH_4_Cl per 1 L of distilled water). Yeast cells grown at 28 °C for 48 h in YPG medium (20 g glucose, 20 g peptone, 10 g yeast extract per 1 L of distilled water) were harvested by centrifugation at 10,000 rpm for 10 min and then resuspended in sterile Ringer’s solution. Next, 100 µL of living cells of the antagonist yeasts (5 × 10^8^ cells per ml) and 100 µL of a 10-day-old culture spore suspension (5 × 10^6^ spores per ml) of a pathogen in Ringer’s solution were transferred to 10 mL tubes containing 4.8 mL wort broth medium. As a control, 100 µL of pathogen spore suspension was added to 4.9 mL medium. Then, the tubes were incubated at 25 °C on a rotary shaker at 150 rpm for 20 h. After this time, in vivo preparations were made by applying a drop of the co-culture to a Thoma counting chamber. The number of germinated spores per one hundred observed was counted in three replications.

### 4.5. Production of Volatile Organic Compounds

**Figure 8:**
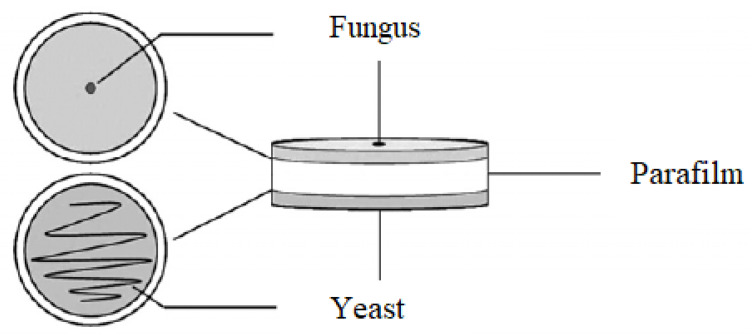
Experimental design to test the antifungal activity of volatile organic compounds produced by yeasts.

The effect of the volatile organic compounds produced by the selected yeasts antagonistic against the phytopathogenic fungi *F. culmorum*, *F. graminearum* and *F. poae* was assessed on Petri dishes with malt extract agar. A thin layer of medium was poured over both parts of a sterile Petri dish and allowed to solidify under a UV lamp. After the medium had solidified the bottoms of the plates were inoculated centrally with a 2-day-old culture of each selected yeast isolate. The opposite sites of the plates were inoculated centrally with a 7-day-old culture of pathogen discs (5 mm in diameter). The Petri dishes were sealed using Parafilm (Figure 8). The control was a Petri dish on which physiological sodium chloride solution was applied instead of the yeast. After 7 days of incubation at 28 °C, the diameter of the mycelium was measured. Based on the measurements, the percentage inhibition of pathogen growth by yeast volatile organic compounds as compared to the control was calculated. Every individual experiment was triplicated.

### 4.6. Enzymatic Activities

Chitinase and β-1,3-glucanase activities were determined using the method described by Kordowska-Wiater et al. [47]. Yeasts were cultivated in the medium composed of 5 g of CWP of each pathogen, 3 g yeast extract, 5 g (NH_4_)_2_SO_4_, 5 g KH_2_PO_4_ per 1 L of distilled water. CWP of *F. culmorum*, *F. graminearum* and *F. poae* were prepared according to the methodology reported by Chan and Tian [43]. Yeast cells were transferred with a sterile inoculation loop to 100-mL flasks containing 30 mL of culture media and incubated at 28 °C on a rotary shaker at 150 rpm for 7 days. Culture filtrates from each flask were taken and centrifuged at 10,000 rpm for 15 min after 3, 5 and 7 days of incubation. The supernatants were used for the analysis of β-1,3-glucanase, chitinase and protein contents.

#### 4.6.1. Production of Chitinase 

The chitinase activity was assayed by measuring the reducing sugars released from colloidal chitin (Sigma-Aldrich, Poznań, Poland). In this assay, 0.5 mL of the resulting supernatant and 0.5 mL of colloidal chitin were added to 10 mL reaction tubes. The mixture was incubated at 50 °C for 60 min to allow the enzymatic reaction to occur. The amount of the reducing sugars released into the reaction medium was determined by the colorimetric method according to Miller [48] using the DNS reagent. Then the absorbance was measured (relative to the control) at λ = 550 nm. One unit of chitinase activity was defined as the amount of the enzyme which yielded 1 μmol of glucose equivalent per minute under the reaction conditions. Each treatment had three replications, and the experiments were repeated twice.

#### 4.6.2. Production of β-1,3-glucanase

The β-1,3-glucanase activity was assayed by measuring the amount of the reducing sugars released from laminarin (Sigma-Aldrich, Poznań, Poland), using glucose as a standard. In this experiment, 0.1 mL of post-culture filtrates were incubated with 0.9 mL 1% laminarin solution in acetate buffer (0.1 M, pH 4.8) at 50 °C for 60 min. The reaction mixtures were incubated at 50 °C for 60 min. The amount of the reducing sugars released into the reaction medium was determined by the colorimetric method, as described above. One unit of β-1,3-glucanase activity was defined as the amount of the enzyme that catalyzed the release of 1 μmol of glucose equivalents per minute. Each treatment had three replications, and the experiments were repeated twice.

#### 4.6.3. Bradford Protein Assay

The Bradford protein assay uses the ability of the Coomassie Brilliant Blue dye to bind to proteins through ionic and hydrophobic bonds. An albumin standard curve was prepared by adding 300 µL of a Coomassie Brilliant Blue dye solution to 10 µL of an albumin solution with protein concentrations of 0.125, 0.25, 0.5, 0.75, and 1.0 mg/mL, and then the absorbance was measured (relative to the control) at λ = 595 nm. The protein content in the post-culture filtrates was determined in the same way. The standard curve of albumin concentration was used to calculate the specific activity expressed in units (U) per milligram of protein.

### 4.7. Siderophore Production

The ability of the investigated yeasts to produce siderophores was assessed by the Chrome Azurol S (CAS agar) assay, a universal qualitative method described by Schwyn and Neilands [48,49], with modifications. To solve the problem of the toxicity of CAS to fungi, the protocol modified by Milagres et al. [50] was applied. A layer of wort agar was poured into sterile Petri dishes and allowed to solidify. After solidification, the medium was cut in half, and one part was replaced with freshly prepared CAS agar cooled to 50 °C. The wort agar portion was inoculated with a 2-day-old culture of the test yeast as close to the siderophore detection medium as possible. The control was the yeast *Rhodotorula glutinis* NRRL YB-252, which has proven siderophore production properties. The test was performed in triplicate. Observations were made after 14 days of incubation at 28 °C. Siderophore production was determined qualitatively by assessing the change in the blue color of the CAS agar medium to pale orange, which indicates the presence of iron chelating siderophores from the Fe-CAS complex.

### 4.8. Antagonistic Activity of Yeast Isolates against Fungi Present on Wheat Grain

We determined the degree to which the test yeast inhibited the spore germination and mycelial growth of *F. culmorum*, *F. graminearum* and *F. poae* on wheat grain. Thirty g of wheat grain and 5 mL of distilled water were placed in 100 mL conical flasks and autoclaved at 121 °C for 21 min. Next, the same volume of sterile distilled water was added to the sterile grains, and the mixture was inoculated with 1 mL of a fungal spore suspension (5 × 10^6^ spores/mL) and 5 mL of a suspension of the test yeast (5 × 10^8^ cfu/mL). Control samples were flasks with grains inoculated with 1 mL of fungal spores and 10 mL of sterile distilled water, and flasks with grains inoculated with 5 mL of the test yeast suspension with the addition of 6 mL of sterile distilled water. The experiment was triplicated. After 7 and 14 days of incubation at 28 °C, the effect of the test yeast on the growth of *F. culmorum*, *F. graminearum* and *F. poae* on wheat grain was assessed visually.

### 4.9. Statistical Analysis

The results were analyzed statistically using Statistica 13.3 (Statsoft, Cracow, Poland) and Excel 2016 (Microsoft, Washington, DC, USA). In order to compare the results, a one-way or a multi-factor analysis of variance (ANOVA) was carried out, after first finding the normality of the distribution of the dependent variable in the compared groups, equal to the variances. The significance of differences between group means was determined using Tukey’s post hoc test. All statistical hypotheses were verified at the significance level of *p* < 0.05.

## 5. Conclusions

Wheat grain is one of the basic raw materials used in human and farm animal nutrition, and at the same time one that is the most exposed to contamination by toxic fungi. In this study, we identified yeasts that were able to limit the growth of *F. culmorum*, *F. graminearum* and *F. poae*, which are common pathogens of wheat. There were more yeast isolates which showed antagonistic activity against fungi of the genus *Fusarium* among those isolated from organic cereals compared to the isolates from the conventional cultivation system. Of all the tested yeasts, only *Cryptococcus carnescens* (E22) isolated from wheat ears coming from the organic farming system limited the growth of the mycelium of all three pathogenic fungi tested using the diffusion method. The yeast isolate *Rhodotorula glutinis* (E20), isolated from wheat ears originating from the organic farming system, was the only isolate to show the ability to produce siderophores. Furthermore, the two aforementioned strains exhibited a high specific activity of β-1,3-glucanase compared to the activity of chitinase, and were the best producers of both enzymes. Summing up, the strain E20 showed all the mechanisms of *Fusarium* antagonism studied, and E22, K2 and K10 had similar activities, except for the production of siderophores. The results indicate that the yeast isolated in this study could be used to improve the biological control of fungi of the genus *Fusarium*. More research is needed to develop a yeast-based strategy for effective biological control of pathogenic fungi of the genus *Fusarium*, in order to reduce and replace chemically synthesized fungicides, which are currently dominating the market. 

## Figures and Tables

**Table 1:** Inhibition of *F. culmorum*, *F. graminearum* and *F. poae* mycelial growth in confrontation assay with yeast isolates on malt extract agar at 28 °C for 7 days.

Yeast			Inhibition of Mycelial Growth ^1^		
			*F. culmorum*	*F. graminearum*	*F. poae*
**Culture collection yeast**		C10 *Candida shehatae*	-	+	-
		**C13 *Candida shehatae***	**+++**	**++**	**-**
		**C14 *Candida fluviatilis***	**+**	**+++**	**-**
		C16 *Candida freyschussi*	-	++	-
		C19 *Candida obtusa*	-	+	++
		C22 *Candida lypolitica*	-	+	-
		**C28 *Candida tropicalis***	**+++**	**+**	**-**
		T2 *Trichosporon brassicae*	++	-	+
		T7 *Trichosporon cutaneum*	++	+	-
		S3 *Saccharomyces cerevisiae*	++	+	-
		S15 *Saccharomyces elipsoideus*	+	-	++
		Rh3 *Rhodotorula mucilaginosa*	+	-	-
**Environmental yeast**	**Conventional**	K1	+	+	-
		**K2**	**-**	**+++**	**++**
		K3	-	++	++
		K7	-	+	+
		**K10**	**++**	**+++**	**-**
	**Organic**	E3	-	+	+
		E8	+	-	-
		E12	+	+	-
		E18	-	++	-
		**E20**	**+++**	**++**	**-**
		E7	+	++	-
		E13	+	++	-
		E1	+	+	-
		E6	-	++	-
		**E22**	**++**	**+**	**+++**
		E30	+	+	++

^1^ Inhibition of mycelial growth (zone of inhibition): +++ (≥5 mm), ++ (4–3 mm), + (2–1 mm), - no inhibition of mycelial growth.

**Table 2:** Inhibition of the mycelial growth of *F. culmorum*, *F. graminearum* and *F. poae* by volatile organic compounds produced by seven yeast strains incubated in Petri dishes with malt extract agar at 28 °C for 7 days.

Yeast	Mycelial Growth Inhibition (%)		
	*F. culmorum*	*F. graminearum*	*F. poae*
C13	83.70 ± 0.64	79.44 ± 0.56	8.70 ± 1.16
C14	46.67 ± 1.11	9.26 ± 0.64	58.70 ± 0.85
C28	87.59 ± 0.85	63.52 ± 1.40	38.52 ± 0.64
K2	65.37 ± 0.85	86.11 ± 0.56	68.15 ± 0.64
K10	61.85 ± 0.64	87.67 ± 0.59	80.93 ± 0.85
E20	78.89 ± 1.11	72.59 ± 0.64	68.52 ± 0.85
E22	66.30 ± 0.52	58.33 ± 0.56	70.00 ± 1.11

± Standard deviation.

## Data Availability

The data presented in this study are available on request from the corresponding author.
